# Highly Competitive Reindeer Males Control Female Behavior during the Rut

**DOI:** 10.1371/journal.pone.0095618

**Published:** 2014-04-23

**Authors:** Guillaume Body, Robert B. Weladji, Øystein Holand, Mauri Nieminen

**Affiliations:** 1 Department of Biology, Concordia University, Montreal, Quebec, Canada; 2 Department of Animal and Aquacultural Sciences, Norwegian University of Life Sciences, Ås, Norway; 3 Finnish Game and Fisheries Research Institute, Reindeer Research Station, Kaamanen, Finland; University of Missouri, United States of America

## Abstract

During the rut, female ungulates move among harems or territories, either to sample mates or to avoid harassment. Females may be herded by a male, may stay with a preferred male, or aggregate near a dominant male to avoid harassment from other males. In fission-fusion group dynamics, female movement is best described by the group’s fission probability, instead of inter-harem movement. In this study, we tested whether male herding ability, female mate choice or harassment avoidance influence fission probability. We recorded group dynamics in a herd of reindeer *Rangifer tarandus* equipped with GPS collars with activity sensors. We found no evidence that the harassment level in the group affected fission probability, or that females sought high rank (i.e. highly competitive and hence successful) males. However, the behavior of high ranked males decreased fission probability. Male herding activity was synchronous with the decrease of fission probability observed during the rut. We concluded that male herding behavior stabilized groups, thereby increasing average group size and consequently the opportunity for sexual selection.

## Introduction

For reasons that remain unclear (e.g. [1]), females of polygynous species commonly move among mating groups or territories [2]–[4]. Female ungulates alter their movement patterns during the breeding season [5], [6], often aggregating around the same male or the same place. These changes might be associated with either male or female mating behavior, likely resulting in increased group size [7]–[9], which ultimately increases the intensity of sexual selection [10]. Understanding factors influencing female movement is therefore important to identify which mating behaviors drive sexual selection [11].

Males may increase their mating opportunities by stabilizing their harems [7], [11], whereas females may gain indirect benefits by leaving harems to sample mates [3], [4], [12]. Females may also move to optimize direct benefits by selecting resource-rich territories [13] or by avoiding harassment [11], [14]. Male coercion, female mate choice and harassment avoidance, can individually or concurrently, constrain female movement. The relative importance of these behaviors on female movement has rarely been estimated, despite their potential for enhancing our understanding of the drivers of sexual selection.

In groups with fission-fusion dynamics [15], [16], group sizes are influenced by the relative rates of group splitting and merging events [17]. Accordingly, males may benefit more from increasing group stability than preventing single females from leaving the group, which is not easy to achieve [11]. Avoiding harassment may also increase group stability. Indeed, females may either aggregate to dilute harassment [18] or stay under the protection of the harem holder, i.e. the dominant male [19]. Because females often copy or follow each other’s movement, a female leaving a group to sample mates may induce fission of the group. Once females have chosen a mate, they would stop sampling, and remain with his group which is less likely to split. Therefore, the influence of male or female mating behavior on female movement may best be represented in fission-fusion group dynamics by an index of group stability, which should be negatively correlated with the group’s fission probability.

Coercion and deception are used by males to prevent individual females from leaving their harems [11]. Herding of females, a common behavior in ungulates, is likely more efficient to decrease the fission probability, and increase male reproductive success, than identifying and following individual females. Although males do not specifically herd females in estrous [20], male reproductive success has been shown to strongly correlate with their social rank [14], [21], [22], which is positively correlated with the stability of their groups [23].

Female ungulates are as likely as female birds to choose their mates [14], but the way they evaluate phenotypic quality is unclear. A number of criteria has been suggested, including vocalization [24], antler size [25], horns size [26], body size [27] or male social rank [14]. Male social rank is an integrative measure of phenotypic quality and may correlate with the characteristics females evaluate when sampling males [11], [14]. Two strategies, threshold sampling and Bayesian sampling, predict a lower probability to leave a male of higher phenotypic quality [28], and consequently, a lower fission probability.

Harassment avoidance is expected to influence the behavior of female ungulates during the breeding season [11], [14]. Harassment level can be diluted by increasing group size, and by joining a harem controlled by a highly competitive male [18], [29]. Solitary females are particularly exposed to harassment [30], so that females prefer to remain in a group, decreasing the fission probability. Females may also seek the protection of a dominant male [19], [31] who will chase satellite males away, thereby keeping harassment to a minimum. Satellite males, usually young and low ranked males, are indeed responsible for harassing females, which may occasionally lead to extreme consequences such as death [32].

Reindeer *Rangifer tarandus* are highly sexually dimorphic [33], [34] and exhibit fission-fusion group dynamics [20], [23]. According to sexual selection theory [35], [36], male herding ability (P_1_), female mate choice (P_2_) or harassment avoidance (P_3_) would decrease the group’s fission probability. If males successfully herd females (P_1_) fission probability should decrease with the time dominant males spend herding or in herding-like activities (Table 1). If females choose their mates (P_2_), fission probability should decrease when the group is led by a high rank male as compared to female only groups (i.e. groups without males), and should increase if the group is led by a male of low social rank (Table 1). Finally, we predict that (P_3_) the fission probability should decrease with increasing number of satellite males, and with the level of their involvement in mating-related activities. It should also correlate positively with the time female spend feeding, considered to be the time when they are undisturbed, as a decrease in time feeding may result from harassment (Table 1).

**Table 1:** Predicted relationship trend between the group’s fission probability and the dominant male activity level, its social rank, the number of satellite males and the activity of satellite males and females in the group.

Group’s fission probability	Dominant male matingactivity level		Dominant malepresence			Satellite males		Females
	High rank	Low rank	Highrank	Withoutmales	Lowrank	Number	Matingactivity level	Feedingfrequency
Herding ability (P_1_)*	−	−						
Female mate choice (P_2_)			−	0	+			
Harassment avoidance (P_3_)						−	−	+

“+” and “−” signs represent the predicted direction of the relationship between a variable and the group’s fission probability. Among male rank categories, the signs represent the relative influence level of the variable on the group’s fission probability.

*This prediction was also assessed using the temporal synchrony between herding and the group’s fission probability.

## Materials and Methods

### Area and Study Herd

We studied a semi-domestic herd of reindeer in Kutuharju Field Reindeer Research Station in Kaamanen, Finland (69°N, 27°E) during the 2011 breeding season (September 8^th^ -October 18^th^). The herd, composed of 11 males (from 1.5 to 5.5 years old) and 34 females (from 1.5 to 10.5 years old), was released into the Sinioivi enclosure (13.4 km^2^). We removed from the analysis the first and the last 24 h to avoid the influence of the herd release and roundup. Ten males and 33 females were originally equipped with a Global Positioning System (GPS) Tellus medium collar and the last male was equipped with a GPS collar in the field on October 1^st^. During the season, the collar of one male (ranked 4 in the social hierarchy) did not work and two female collars stopped working on October 1^st^ and October 16^th^, respectively. All GPS collars synchronously recorded their position every 15 minutes, for a total of 3800 recordings. At each recording time *t*, we generated a map of individual positions.

### Ethics Statement

Handling of animals and data collection was done in agreement with the Animal Ethics and Care certificate provided by Concordia University (AREC-2010-WELA and AREC-2011-WELA) and by the Finnish National Advisory Board on Research Ethics.

### Group Definition

We defined groups from the spatial aggregation of individuals. We used a chain rule based on the nearest neighbor distance [37], [38] stating that two neighbors belong to the same group if their inter-individual distance was below 89 m (see Method S1 and Fig. S1 in the Supporting Information for details). Then, we followed each group (≥2 females and ≥0 male) until it disappeared. A group could disappear if it split (fission) or merged with another one (fusion). Male and individual female departures and junctions from the group or to the group did not influence the group identity. To prevent registering excessive splitting events due to GPS errors or GPS location failures (i.e. missing data), we applied a smoothing procedure to the group identity. Any reversion, i.e. a group splitting followed by the sub-groups merging together [39], which lasted less than 30 min was disregarded and the same group identity was subsequently used. Because small groups appeared particularly sensitive to GPS errors, we increased this time up to 60 min for groups containing only two females. For descriptive purpose, we also assessed the number of groups present in the enclosure every 25 hours (to insure data independence) as well as their individual duration. We report the average number of group and their half-life (i.e. the median group duration) according to the period of rut and the social rank of the dominant male (see definitions below).

### Survival Analysis

#### Model

We ran a non-parametric survival analysis model (a Cox model with the coxph function using the package “survival” in R, [40]) with the duration of the group as index of survivorship (for similar analysis, see [41]). As we were interested in the group’s fission probability, we recorded splitting events as death events, whereas merging events were recorded as censoring events. Indeed, the group had not split when the fusion happened, but it cannot be followed further as its composition dramatically changed.

#### Explanatory variables

We included the following variables in the full model according to our predictions (P_1_, P_2_, P_3_): the social rank (see below) of the dominant male (Male; P_1_, P_2_) and the proportion of time it spent in mating-related activities (DomAct; P_1_); the number of satellite males in the group (NbSat; P_3_), and the proportion of time they spent in mating-related activities (SatAct; P_3_); and the percentage of time females spent feeding (FemEat; P_3_). We also included two covariables: the group size (GpSize), as larger groups are expected to split more easily [39], and the period of the rut (Period) (see below) as preliminary analyses revealed temporal variability of group dynamics. We had, unfortunately, no data to control for the possible influence of habitat structure [41]–[43]. However, it is unlikely that habitat selection varied during the breeding season in a way that would influence the reported results.

We classified males (*Male*) based on their social rank (a measure of their quality *sensu* [28]). We established a linear hierarchy among males from field observations of agonistic behaviors. Because male ranked 4 was not followed by GPS, the top three males were classified as “high rank” and the remaining eight males as “low rank”. This threshold is based on field observations as the three top ranked males were most often seen holding a harem. Moreover, this classification enhanced statistical power (as some “low rank” males were still able to lead medium size groups), and was related to body mass and antler size. Indeed high rank males weighted more than 125 kg and their antlers measured more than 85 cm, while low rank males were lighter than 115 kg and their antlers were smaller than 85cm. The variable *Male* included a third class (“without male”) for female-only groups. In this paper, “high/low rank” refers to the linear hierarchy among males in the entire herd; while “dominant/satellite” refers to the social status within each group. Because of the strong correlation between social rank and both body mass and antler size, high rank males are generally highly competitive.

The breeding season was divided in two periods (*Period*). The rutting period was defined as the peak rut week and the early peak rut week [44] for a total period of two weeks (September 23^rd^ to October 6^th^), when mating behaviors were more frequent. The time before and the time after the rutting period, were considered as “outside rut”. Groups were ascribed to a given period based on the average date of the group (Eq. 1).
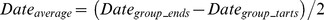
(1)


We determined the median group composition from GPS records. *GpSize* was consequently the median number of females in the group and *NbSat* the median number of males in the group, excluding the dominant male. We assumed that the male with the highest social rank in the group was the dominant male. When the dominant male changed during the duration of the group, we removed the group from analysis.

The percentage of time males or females spent in a given activity was estimated from the activity sensor records using the recursive model [45] at each recording *t* (see Method S2 and Fig. S2 in Supplementary Information for details). Once resting periods were detected (Fig. S3, Fig. S4), we estimated during the active periods the percentage of time males spent in mating-related activities (Fig. S2), i.e. standing, walking or running which represent short behaviours such as chasing males, herding females, threatening, grunting, courting, seeking copulation and being vigilant toward other males [46]. The average percentage of time the dominant male spent in mating-related activities in the group formed the *DomAct* variable. We used the average of the cumulative percentage of time satellite males spent in mating-related activities to form the *SatAct* variable. Similarly, we estimated the average percentage of time active females spent feeding in the group at each recording *t* (Fig. S2), and we averaged these values throughout the duration of the group to form the variable *FemEat*.

#### Model selection

The most complex model fitted to explain the group’s fission probability included Period, Male, GpSize, NbSat, DomAct, SatAct and FemEat, and a number of interactions among those variables. We included a two-way interaction Male:Period to take into account the fact male characteristics influence the timing of their mating behavior [46]. Within these different periods, male’s characteristics can also influence the efficiency of his mating behaviours or his ability to manage a larger group. Consequently, we included the three-way interactions Male:Period:DomAct, and Male:Period:GpSize. We performed all possible subsets of models [47] and extracted the Akaike Information Criteria (AIC) from each [40]. The number of different possible models, 488, was lower than the sample size (see results) as preconized [48]. We calculated AIC weights for each variable from all subsets [47], but we only displayed models with a ΔAIC≤2. Then, we selected, among these models, the one including the variables with the highest relative importance (obtained by summing AIC weights; [49]) for both graphical purpose and effect sizes, which dealt with model uncertainty [47].

### Temporal Synchrony

#### Temporal variation in herding frequency

To obtain a more precise measure of herding, we used a long-term dataset (15 years, from 1996 to 2011, without 1998) of direct observations of dominant male behavior during the rut season to assess the synchrony between herding and group’s fission probability. Behavioral records were collected using a 15 min focal observation method [46]. As herding a female regularly switched to a chase [50], we summed behaviors classified in the field as either “herd” or “chase female” to assess the frequency of the herding behavior. We modelled the proportion of time spent herding as a function of the number of days to the beginning of the peak rut using a generalized additive model (GAM), with a smoothing parameter k of 8. The beginning of the peak rut was calculated for each year by the back-dating procedure (as described above for the variable *Period*) and all years were then pooled together.

#### Temporal variation of the group’s fission probability

Using the above GPS dataset recorded in 2011, we calculated the group’s fission probability at each recording time *t* as the proportion of group at time *t_–1_* that split at time *t*. We analyzed the temporal variability of the fission probability using a GAM with the time as explanatory variable, with a smoothing parameter k of 8. We included the mean group size as covariate. The GAM had a binomial link and data were weighted by the number of groups at time *t_–1_*.

## Results

### Number of Groups and Group Half-life

**Figure 1:**
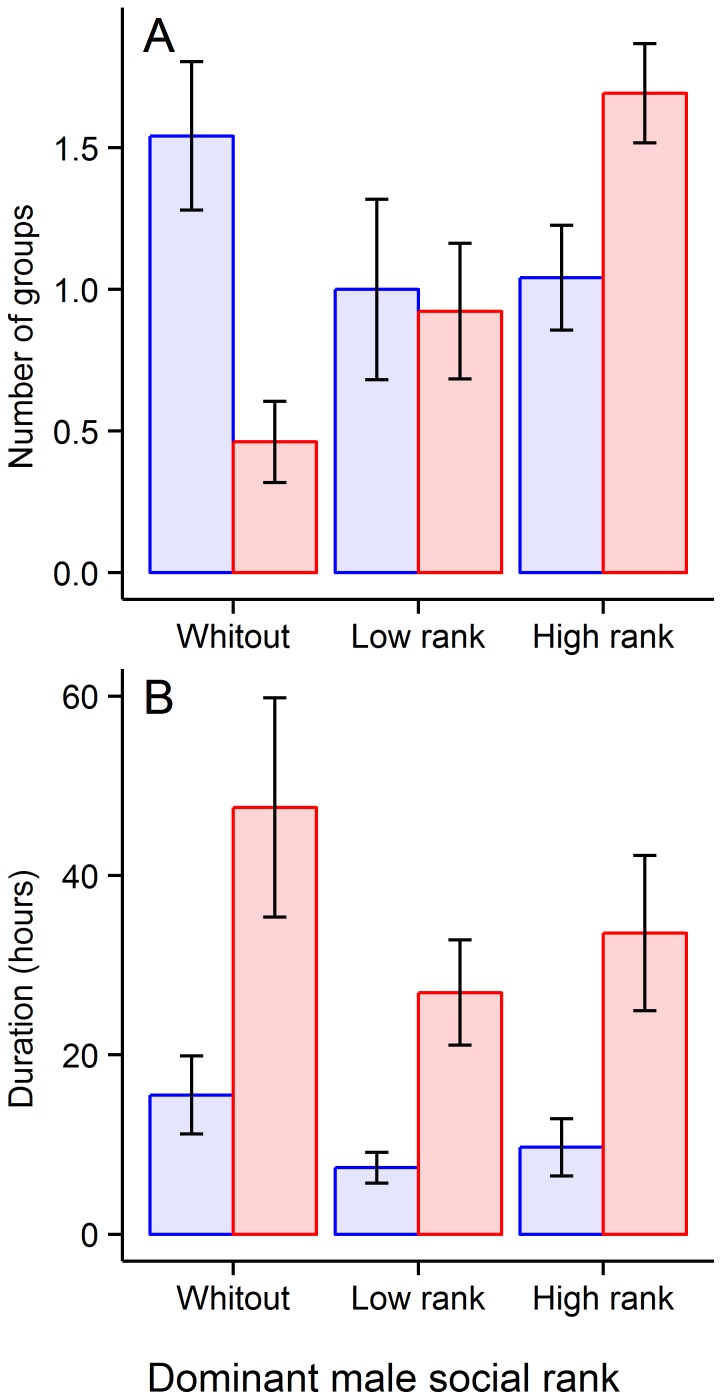
Number of groups (A) and their half-life (B) according to the social rank of the dominant male and the period of the rut. Averages are represented in each category with their standard errors. Left-blue bars and right-red bars correspond to the outside rut and during rut periods, respectively.

Outside the rut period, there were on average (± SE) 1.5±0.3 groups without males, 1.0±0.3 groups with a low rank male, and 1.0±0.2 groups with a high rank male (Fig. 1A). The median duration of these groups were respectively 15.5±4.3 hours, 7.4±1.7 h and 9.7±3.1 h (Fig. 1B). At any time during the rut, there were 0.5±0.1 groups without males, 0.9±0.2 with a low rank male and 1.7±0.2 with a high rank male (Fig. 1A). These groups lasted on average 47.6±12.2 h, 27.0±5.9 h, and 33.4±8.7 h, respectively (Fig. 1B).

### Survival Analysis

We analyzed 1075 groups which included 335 splitting events. Among these groups, 879 were recorded outside the rut period (N = 300, 276 and 303 without males, with low rank and high rank males, respectively), whereas 196 were recorded during the rut period (N = 42, 60 and 94 without males, with low rank and high rank male, respectively). Model certainty to explain the group’s fission probability was low, as it took 166 models to reach 0.95 of the AIC weights. Twelve models had ΔAIC≤2 (Table 2) and they represented together 0.31 of the AIC weights. Confidence in variable selection was high (Table 2), as the variables *Period*, *Male*, *DomAct*, and *GpSize* had AIC weights over 0.95, while *SatAct*, *NbSat*, and *FemEat* had AIC weights≤0.45. The three interactions formed with the variable *Male* (*Male:Period*; *Male:DomAct*; *Male:GpSize*) had high AIC weights (≥0.64, Table 2), while the other interactions had AIC weights≤0.46 (Table 2). The model 1 (i.e. with the lowest AIC) in Table 2 was the combination of the two most parsimonious models (models 4 and 11, Table 2), and included all the variables with high AIC weights, in contrast to models 4 and 11. Therefore, model 1 was the best model to represent AIC weights of the different variables, and it was used for interpretation hereafter.

**Table 2:** Selection of the model explaining variations of the group’s fission probability.

Candidate models								Interaction between *Male* and:			Interaction between *GpSize* and:						
variable	*Period*	*GpSize*	*Male*	*DomAct*	*NbSat*	*SatAct*	*FemEat*	*:Period*	*:DomAct*	*:both* 1	*:Male*	*:Period*	*:both* 2	AIC	ΔAIC	AIC_w_	acc AIC_w_
w_i_	0.99	∼1.00	∼1.00	0.96	0.34	0.45	0.33	0.65	0.84	0.07	0.81	0.46	0.15				
1	×	×	×	×				×	×		×			3930.9	0	0.044	0.044
2	×	×	×	×		×		×	×		×			3931.3	+0.41	0.036	0.081
3	×	×	×	×				×	×		×	×		3931.4	+0.54	0.034	0.115
4	×	×	×	×					×		×			3931.4	+0.56	0.034	0.149
5	×	×	×	×		×			×		×			3932.0	+1.12	0.025	0.174
6	×	×	×	×		×		×	×		×	×		3932.1	+1.20	0.024	0.199
7	×	×	×	×	×	×		×	×		×			3932.3	+1.37	0.022	0.221
8	×	×	×	×			×	×	×		×			3932.3	+1.42	0.022	0.243
9	×	×	×	×	×			×	×		×			3932.6	+1.73	0.019	0.262
10	×	×	×	×		×	×	×	×					3932.7	+1.83	0.018	0.280
11	×	×	×	×				×	×					3932.8	+1.93	0.017	0.297
12	×	×	×	×	×	×		×	×		×	×		3932.9	+1.97	0.017	0.313

We represented the variables included in the 12 best models that have ΔAIC≤2, with their respective AIC values, ΔAIC, their AIC weights (AIC_w_) and the cumulative sum of the AIC weights (acc AIC_w_). We also present the cumulative sum of the AIC_w_ in which each variable is presented, giving the variable’s AIC weight (w_i_; in line).

1 interaction: *Male:Period:DomAct*.

2 interaction: *Male:Period:Gpsize*.

**Figure 2:**
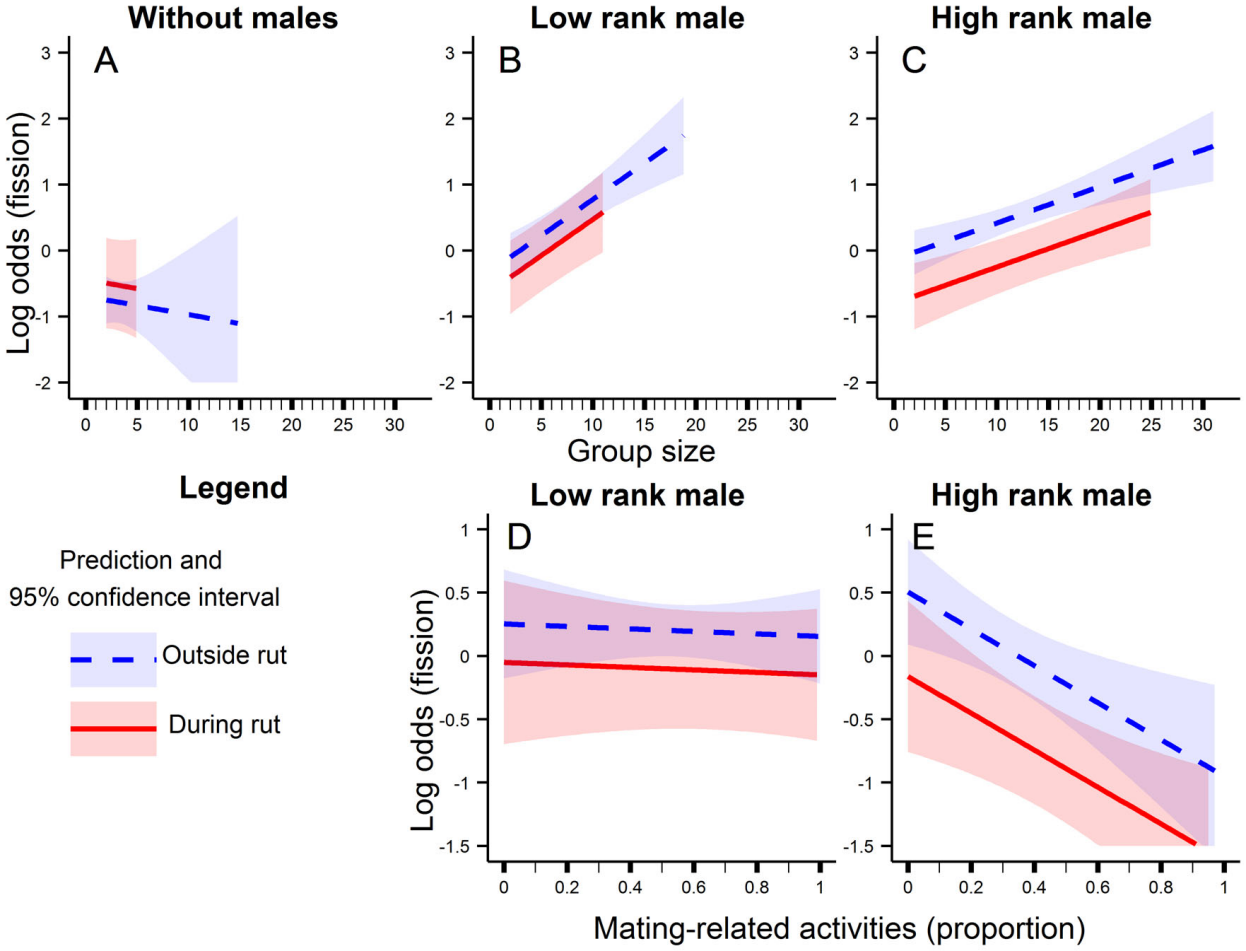
Social and behavioral influence on group’s fission probability. Partial effect on group’s fission probability of the group size (A, B, C) and of the proportion of time the dominant males spent in mating-related activities (D, E) according to the social rank of the dominant male of the group : without males (A), low rank male (B, D), high rank male (C, E), and according to the period of the rut: outside the rut (continuous and blue lines) and during the rut period (dashed and red lines). Effects are presented with their 95% confidence intervals.

The variables related to harassment avoidance, i.e. *NbSat*, *SatAct* and *FemEat*, did not influence the group’s fission probability, which was independent of group size when males were absent (Table 3, Fig. 2A), and increased with group size when the dominant male was of low (Table 3, Fig. 2B) or high (Table 3, Fig. 2C) rank. The fission probability was lower in absence of males (Fig. 2A), than in their presence, regardless of their rank (Fig. 2B, C). The mating-related activities of low ranked dominant males did not influence the fission probability (Table 3, Fig. 2D). Conversely, the proportion of time high ranked dominant males spent in mating-related activities decreased the fission probability (Table 3, Fig. 2E). As expected, the fission probability decreased during the rut period, especially for high rank males (Table 3, Fig. 2B vs. Fig. 2C, Fig. 2D vs. Fig. 2E). The model explained about 8% of the variability in the group’s fission probability (R^2^ = 7.8%), and the model discrimination power had a concordance value of 63.7% ±2.1.

**Table 3:** Parameter estimates and corresponding standard error (SE) of the final model explaining the fission probability of groups without males (A), groups controlled by a low rank dominant male (B), and groups controlled by a high rank dominant male (C).

	(A) Without males		(B) Low rank male		(C) High rank male	
	Estimates ± SE	p-value	Estimates ± SE	p-value	Estimates ± SE	p-value
Intercept	0		0.03±0.39	p = 0.930	0.55±0.36	p = 0.130
Period	0.26±0.36	p = 0.48	−0.30±0.27	p = 0.270	−0.67±0.21	p = 0.002
Group size	−0.03±0.07	p = 0.69	0.11±0.02	p<0.001	0.06±0.01	p<0.001
Dominant malesexual activity			−0.10±0.35	p = 0.780	−1.46±0.50	p = 0.003

### Temporal Synchrony

**Figure 3:**
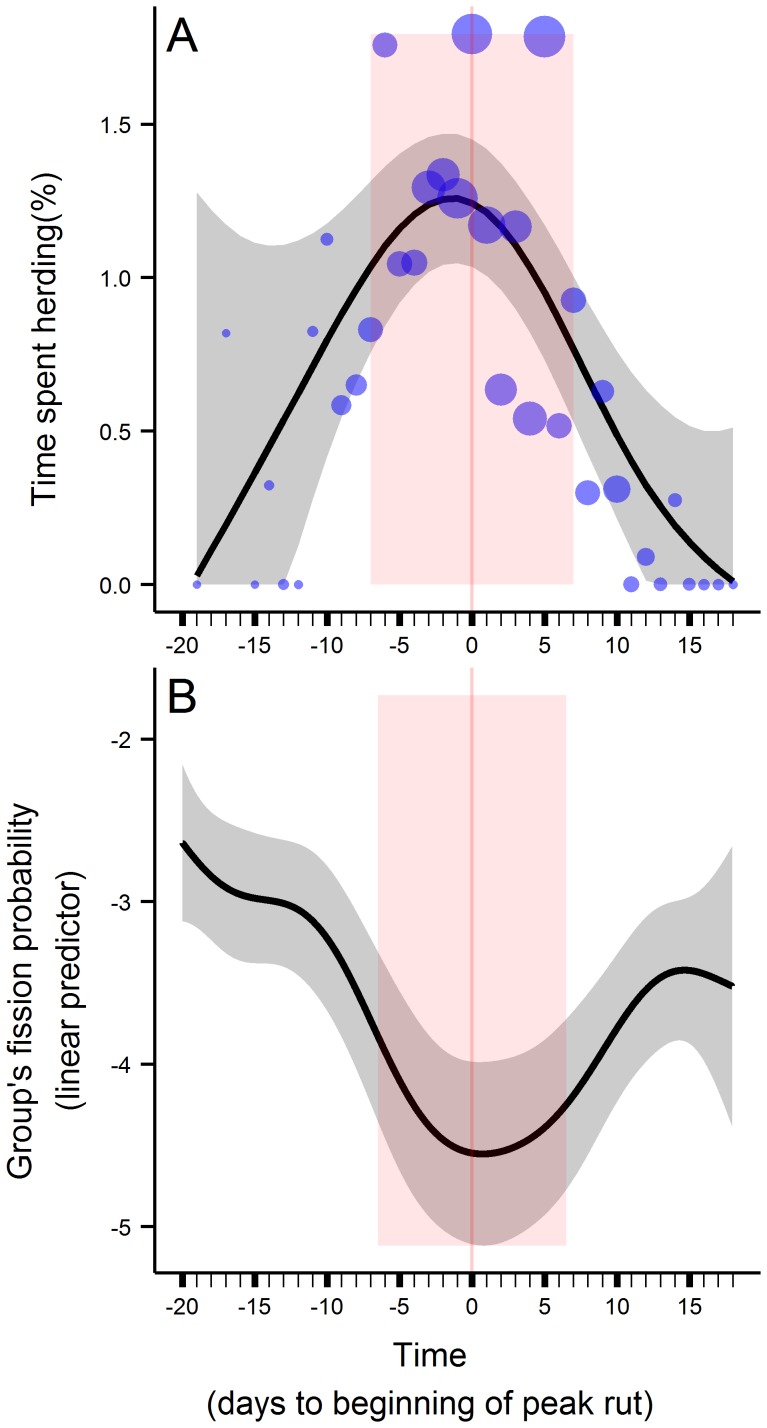
Temporal variations of herding frequency and group’s fission probability. Temporal variations in the herding frequency of dominant males (A), and in the group’s fission probability (B). Black lines represent the predictions and the grey areas surrounding them are their 95% confidence intervals. The red vertical bands represent the period during the rut (“peak rut week” versus “outside rut”), the darker red line the beginning of the peak rut week. Blue dots in panel A are the observed daily average of the time spent herding by dominant males, and their sizes are proportional to the number of observations.

The beginnings of the peak rut ranged from September 29^th^ to October 13^th^ depending on the year. All years pooled together, behavioral observations happened from 19 days before the beginning of the peak rut to 26 days after (N = 853). The percentage of time spent herding varied throughout the mating season (p<0.001), displaying a dome shape with a maximum at the beginning of the peak rut (Fig. 3A).

The group’s fission probability varied throughout the mating season (p<0.001), displaying an inverse dome shape with a minimum at the beginning of the peak rut (Fig. 3B). In addition, the mean group size increased the group’s fission probability (slope ± SE = 0.16±0.02, p<0.001).

## Discussion

During the breeding season, males may try to increase their mating opportunities by herding females into their harem, and females may continuously be on movement to sample mates, thereby influencing mating groups size, and hence the opportunity for sexual selection [10]. In this study, we estimated the relative influence of male and female mating tactics on females’ movement, using a herd of reindeer exhibiting fission-fusion group dynamics that we followed using GPS. Our results only supported the prediction about male herding ability (P_1_), as we found the level of mating-related activities of highly competitive males to decrease group’s fission probability and that, temporal variations of both herding and group’s fission probability were exact opposites. Contrary to predictions, we found no evidence for female mate choice (P_2_), or for harassment avoidance (P_3_).

As males herded females, there was a tendency for groups to be more stable. The resulting decrease in fission rate, induced an increase in average group size [17]. Larger harems retain more estrous females [23]. Consequently, more efficient is the herding, the greater the number of estrous females a male can have in his harem, depleting mating opportunities from his competitors, and consequently increasing the opportunity for sexual selection [10]. This process is reinforced by the difference in herding ability among males. Low rank males are inefficient herders either due to their lower body condition [51] or their inexperience for the youngest ones [31], [44], [52]. Herding is expressed mostly at the beginning of the peak-rut. This suggests that dominant males focused their attention, during the peak rut week or toward the end of the peak rut, on other mating behaviors such as courting or tending females. Together with interference competition, which happened through fights for dominance when groups merged together [53], herding provides an additional mechanism to explain high sexual selection in reindeer.

We found no evidence that female mate choice influenced the group’s fission probability as groups without males were less likely to split than groups with males. However, females are known to change their behavior during a short period of time around the estrous [1], [5], [6], to be choosy only during their estrous [54]. Females may also express their choice through quick behavior, such as joining satellite males outside the harem [4] or vocalizing when approached by satellite males [19] to increase agonistic interactions among males. Therefore, we may have to focus more on the estrous period to improve our understanding of the role of mate choice on female ungulates’ movement. Moreover, we argue that the question “why females stay within a group” [3], addressed also in this study, is as important as the question “where are females going” [1], [4]. Female mating tactics are also highly variable among individuals, being experience- and condition dependent [4]. Therefore, it might be easier to detect female mate choice when studying individual behavior, rather than group behavior as we did in this study.

Variables representing harassment had a low statistical support in explaining the fission probability. The increase of fission probability with increasing group size is also inconsistent with the dilution effect of harassment (as observed in red deer *Cervus elaphus*_,_ [18]). Harassment level may, however, be more intense when females are solitary [30], given also that females prefer to be with other females [55], [56]. Consequently, females might only lessen the costs of harassment by avoiding being solitary. This is in accordance with earlier findings that the number of solitary females decreased during rut [7], [57].

A recent conceptual framework [15] hypothesized that social relationships are important in determining group stability. Our results validate this hypothesis as social environment (group size, presence of males, male characteristics) and social behavior (herding) influenced group’s fission probability. Although herding behavior seems to be attributed to dominant males during the breeding season, the increase of the group’s fission probability with group size is not season-specific [39]. Indeed, both group size and presence of males decrease the level of synchrony in activity among individuals [58], [59], a key factor explaining group cohesion [60], [61]. The resulting negative correlation between group size and group cohesion could be reversed if the relative benefits expected from sociality (i.e. staying in a cohesive group) outweigh the benefits expected from reaching a desired patch [62]. In this predator-free reindeer herd, females maintain weak social bonds [63], the group size does not decrease the harassment level, and food patches are widely dispersed. Consequently, there are few benefits expected from social cohesion which may explain the high fission rate observed.

Our study contrasted the relative effect of male and female mating behaviors in a highly sexually dimorphic ungulate, and clearly showed that highly competitive males, through herding and other mating-related activities, strongly influence females’ movement pattern. While studies of female mating tactics are needed in mammals [14], we advocate to concurrently evaluate hypotheses derived for both sexes, as sexual coercion is frequent [11], and female choice may be more apparent than real, a lesson learnt from primates [64].

## Supporting Information

Figure S1 **Observed and simulated nearest-neighbor distance.** Observed (bold dashed line) and simulated (bold continuous line) cumulative distribution function of the nearest-neighbor distance (G function) with their 90% confidence intervals. G(r) represents the proportion of the individuals in the population (y-axis) that has their nearest-neighbor within the distance r (x-axis). We display the difference between the two confident intervals at the estimated intra-group maximal distance (r_max_ = 89 m).
(JPG)

Figure S2 **Activities probability according to activity sensor records.** Relationship between the left-right (*X_adj_*), the forward-backward (*Y_adj_*) movements of the activity sensor and the proportion of time spent resting for females (A), and males (B), and of the proportion of time spent feeding for females (C), and in mating-related activities for males (D). The darkness of each square is proportional to the observed number of data with the corresponding [*X_adj_*,*Y_adj_*] adjusted values.
(TIF)

Figure S3 **Steps of the estimation of the resting bouts.** We estimated the proportion of time spent resting from the recursive model (A), then we applied a threshold at 0.6 (red line) to obtain binary resting time (B). We applied a smoothing procedure to clearly identify resting bouts (top layer, C). The calculation of the proportion of time spent feeding for females only applied to records of an active (i.e. excluding “resting”) period (i.e. the bottom layer).
(TIF)

Figure S4 **Duration of the resting bouts.** The vertical red line correspond to the smallest duration of the resting bouts (i.e. 45 min) used in the exploratory variables.
(TIF)

Method S1 **Estimation of the maximal distance among neighbors of the same group.**
(DOCX)

Method S2 **Estimation of activity levels from activity sensors.**
(DOCX)
