# Supplementary figures and images for: Highly Competitive Reindeer Males Control Female Behavior during the Rut
Source: PLoS One. 2014 Apr 23;9(4):e95618. doi: 10.1371/journal.pone.0095618 (PMC3997419; doi:10.1371/journal.pone.0095618)

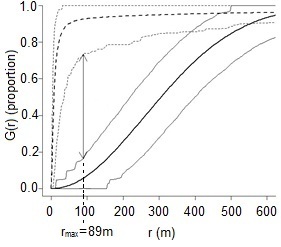

Supplement: Figure S1 — Observed and simulated nearest-neighbor distance. Observed (bold dashed line) and simulated (bold continuous line) cumulative distribution function of the nearest-neighbor distance (G function) with their 90% confidence intervals. G(r) represents the proportion of the individuals in the population (y-axis) that has their nearest-neighbor within the distance r (x-axis). We display the difference between the two confident intervals at the estimated intra-group maximal distance (rmax = 89 m). (JPG) [file pone.0095618.s001.jpg]

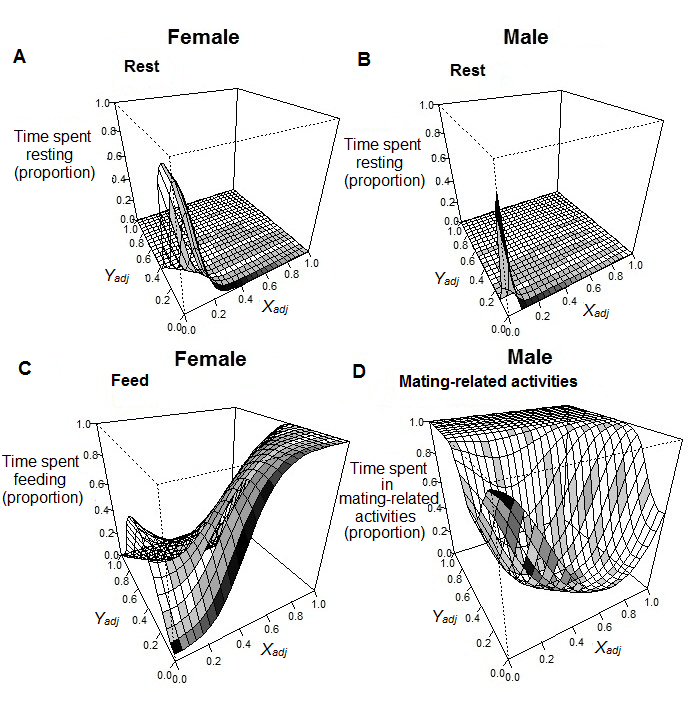

Supplement: Figure S2 — Activities probability according to activity sensor records. Relationship between the left-right (Xadj), the forward-backward (Yadj) movements of the activity sensor and the proportion of time spent resting for females (A), and males (B), and of the proportion of time spent feeding for females (C), and in mating-related activities for males (D). The darkness of each square is proportional to the observed number of data with the corresponding [Xadj,Yadj] adjusted values. (TIF) [file pone.0095618.s002.tif]

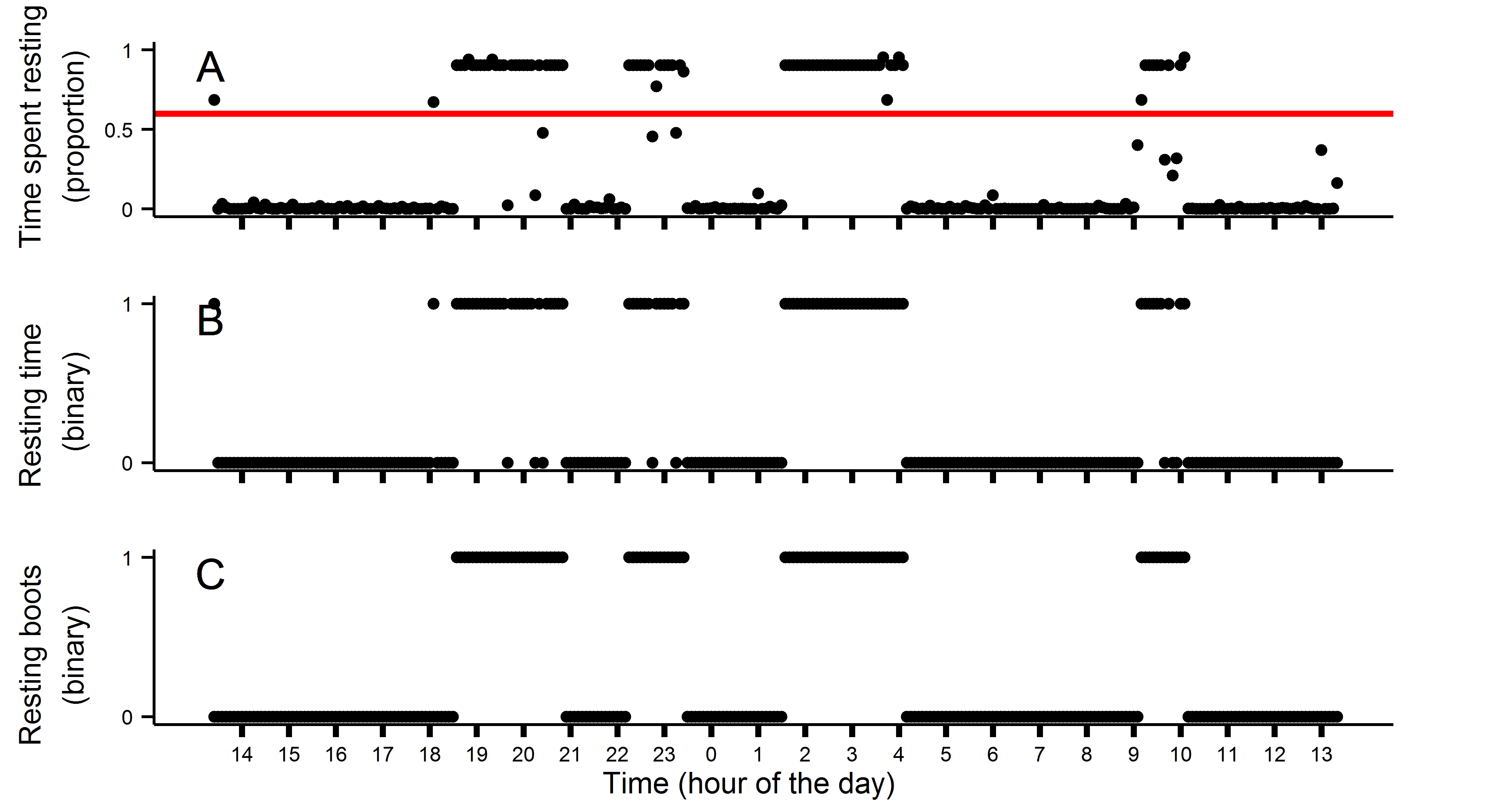

Supplement: Figure S3 — Steps of the estimation of the resting bouts. We estimated the proportion of time spent resting from the recursive model (A), then we applied a threshold at 0.6 (red line) to obtain binary resting time (B). We applied a smoothing procedure to clearly identify resting bouts (top layer, C). The calculation of the proportion of time spent feeding for females only applied to records of an active (i.e. excluding “resting”) period (i.e. the bottom layer). (TIF) [file pone.0095618.s003.tif]

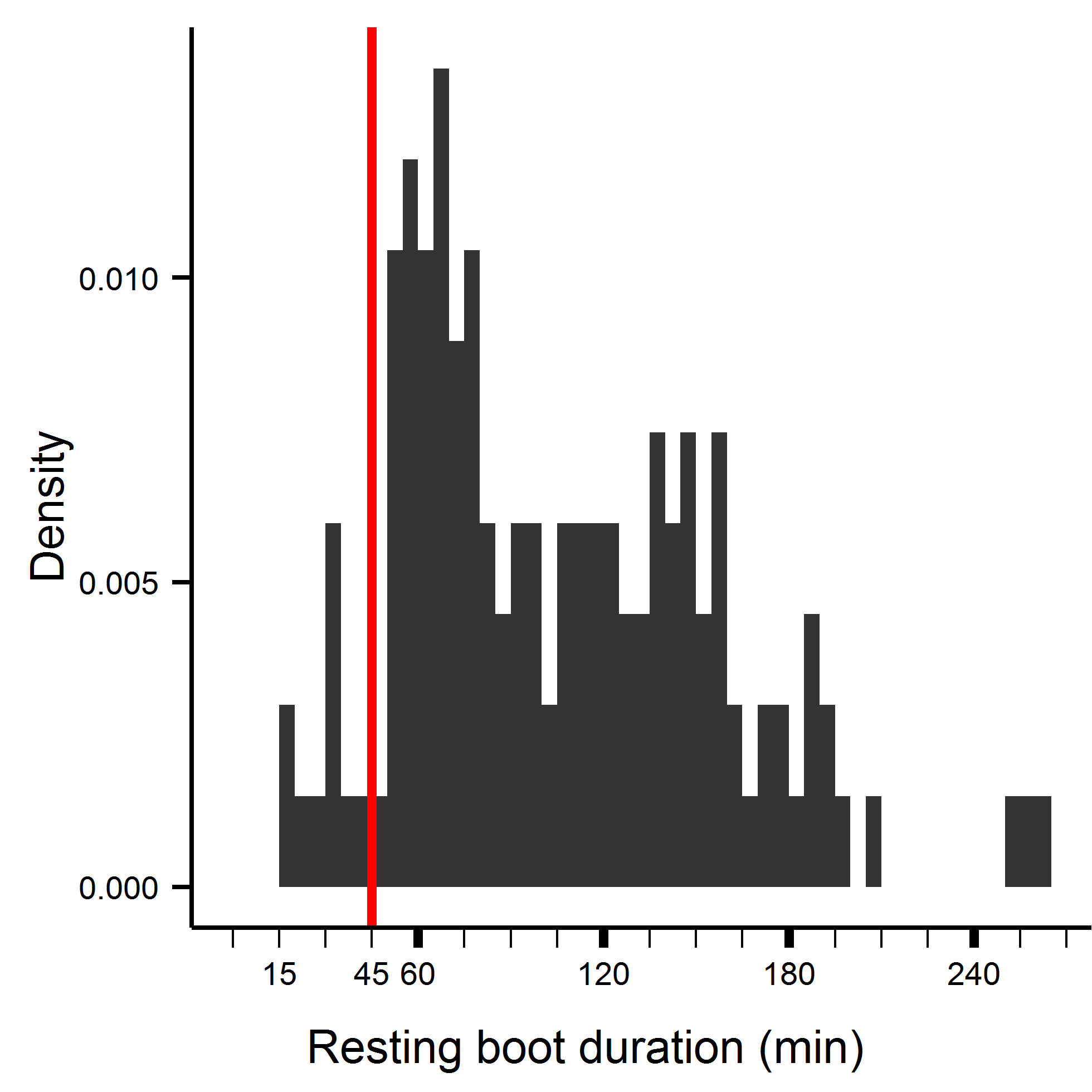

Supplement: Figure S4 — Duration of the resting bouts. The vertical red line correspond to the smallest duration of the resting bouts (i.e. 45 min) used in the exploratory variables. (TIF) [file pone.0095618.s004.tif]
