# Supplementary material for: Highly Competitive Reindeer Males Control Female Behavior during the Rut
Source: PLoS One. 2014 Apr 23;9(4):e95618. doi: 10.1371/journal.pone.0095618 (PMC3997419; doi:10.1371/journal.pone.0095618)
Supplement: Method S1 — Estimation of the maximal distance among neighbors of the same group. (DOCX) [file pone.0095618.s005.docx]

## Method S1 Estimation of the maximal distance among neighbours of the same group

To estimate the maximal distance that neighbours can maintain within a group (r_max_), we performed a spatial analysis on the point patterns obtained during our 2011 study period, only using times during which every GPS collars recorded their positions (i.e. with 42 data points). For each time *t*, we calculated the cumulative distribution function of the nearest-neighbour distance (the G function [1]) with edge corrected by the Kaplan-Meier method (referred below as the G_km_(r) function, where r is the distance to the nearest-neighbour in meters [2]). To do this, we used the “Gest” function from the Spatstat package in R [3]. We calculated for each curve the distance “r*” at which the observed curve was the most different to the upper limit of the 95% confidence envelope (one sided) of the theoretical G_km_(r) function. This envelope was generated by a Monte-Carlo procedure. We generated 1000 times 42 random points in our enclosure and we calculated their G_km_ functions. We defined the one sided confident interval as follow: the lower limit was equals to 0 for any r; and for each r value, the upper limit was defined by the 50^th^ (5%) highest value of the G_km_(r) function. We selected “r_max_” as the 95^th^ upper percentile of the r*. Therefore, r_max_ represents the distance to the nearest neighbour from which 95% of the most aggregated pattern can be estimated.

The data set from 2011 included 2863 recording times (75.3% of the whole dataset). The observed G_km_(r) functions showed a significant aggregated pattern below 463 m (based on the intersection between the average of the Gest function and the upper limit of the 95% confidence intervals of the simulated G_km_(r) function; Fig. S1) and our r_max_ was estimated to be 89 m.

**References**

1. Ripley, B D (1988) Statistical inference for spatial processes. New York: Cambridge University Press.

2. Baddeley A, Gill RD (1997) Kaplan-Meier Estimators of Distance Distributions for Spatial Point Processes. Ann Stat 25: 263–292.

3. Baddeley A (2010) Analysing spatial point patterns in R. CSIRO: version 4.1. Available: http://www.csiro.au/files/files/piph.pdf.
