# Supplementary material for: Highly Competitive Reindeer Males Control Female Behavior during the Rut
Source: PLoS One. 2014 Apr 23;9(4):e95618. doi: 10.1371/journal.pone.0095618 (PMC3997419; doi:10.1371/journal.pone.0095618)
Supplement: Method S2 — Estimation of activity levels from activity sensors. (DOCX) [file pone.0095618.s006.docx]

**Method S2.** **Estimation of activity levels from activity sensors**

We estimated the proportion of time spent feeding for females and the proportion of time spent in mating-related activities for males during the active bouts of time (i.e. excluding “resting time”) using the recursive model [1]. During the rut, running, walking and standing are associated with male mating activities such as chasing males, herding females, threatening, grunting, courting, seeking copulation and being vigilant toward other males. In contrast, feeding is unrelated to mating activities [2].

Methods

*Activity level*

The dataset for the validation of the activity sensor was composed of a dataset previously reported [1], in addition to the behavioural observation collected in the field during the 2011 rut season (i.e. additional 39 data for males, 133 data for females in similar condition with the Group B dataset; see [1]). We used 80% of the dataset as training dataset to estimate the equation; then we assessed the quality of these models by calculating the bias and spread (see details in [3]; and an application example in [1]) from the remaining 20% (the testing dataset). The final equations were fitted on the whole dataset.

We proceeded in three steps to estimate the proportion of time spent feeding for females, and the proportion of time spent in sexual activities for males: 1) we estimated the proportion of time spent resting for each record; 2) from these estimations, we applied a smoothing procedure to obtain bouts of resting time; 3) we fitted the activity equations on the data that were not included in a resting period (see “estimation of resting period” section, below for an example of these three steps).

Step 1: We estimated the proportion of time spent resting (i.e. rest versus stand/feed/walk/run) for both sexes using a generalized additive model (GAM). As graphical representation of the male equation displayed an obvious error for Y-values (forward-backward movement of the GPS collar) greater than 0.6, adjustment were made on those values.

Step 2: To obtain binary data, we applied a threshold at 60% of time spent resting, which is a conservative measure compared to a threshold at 50%, above which odds are higher to be resting. Due to the variability among collars, some collars were overall estimating low values of the percentage of time spent resting. Therefore, we moved down the threshold to the percentage of time spent resting corresponding to the 90^th^ highest percentile if this one was lower than 60%. This procedure assured to catch the top layer of the estimations of the proportion of time spent resting (see “estimation of resting bouts” section for an easy visual identification of the top layer). Then, we transformed these binary data (rest/not rest) to bouts of resting time. We smoothed the sequential results based on sliding windows of five records centered on the data in focus. If at least four of them agreed with a given value, we attributed this value to the data in focus. Then, we repeated this procedure, but attributing the value of the majority of the five recalculated records to the focal data. This procedure allowed having bouts of resting lasting at least 45 min, i.e. three records. Forty five minutes spent resting allowed us to capture 93% of the resting periods, based on a 5 minutes frequency dataset (see “estimation of resting bouts” section).

Step 3: We estimated the proportion of time spent feeding for females (feed versus stand/walk/run) and the proportion of time spent in sexual activities for males (stand/walk/run versus feed) using a GAM in the recursive model. To set up these equations, we removed from the validation dataset any observation that included resting activity, as step 1 and step 2 removed for the application dataset the occurrence of resting behaviour. We applied these three steps of the procedure to the whole dataset of each individual taken separately.

The mean proportion of time a dominant male spent in sexual activities, disregarding his resting periods, was considered as the *DomAct* variable of that group. At each relocation time *t* we summed the proportion of time spent in sexual activities by satellite males (assuming they would have a cumulative effect on the disturbance level of the group), disregarding their resting periods. The average of these values was considered as the *SatAct* variable of the group. The mean proportion of the time females spent feeding was calculated for each group at each relocation time *t*, disregarding resting females. The average of these values was considered as the *FemEat* variable of the group.

*Estimation of resting bouts*

We determined the duration of resting bouts using data collected from activity sensors during the 2008 rutting season. During this year, one female was equipped with the same Tellus medium GPS collar as the ones used in this chapter. This collar was however set-up to record positions and the activity data every 5 min and recorded the data from September 20th at 00:00 to October 14th 23:55, i.e. 7193 relocation time.

We applied the two first step of the activity estimation described above to determine resting bouts. This procedure smoothed the resting bout for a minimal duration of three recording times. Using the 5 min data frequency, the minimal resting bout for this estimation was 15 min.

Results

*Activity level*

We recorded 479 behavioural observations for females and 389 for males, which were used as training and testing dataset to fit the resting models. The relationship between activity sensor values (*X_adj_* and *Y_adj_*) and the predicted proportion of time spent resting are represented for females (Fig. S2A) and for males (Fig. S2B). Both models had no bias (p = 0.959 for females; p = 0.191 for males), and had a weak spread (p = 0.001, slope = 0.86 for females; p < 0.001, slope = 0.78 for males; a slope of 1 would mean no spread). The dataset used for fitting the feeding (females) and sexual activity (males) models were composed of 365 (females) and 309 (males) behavioural observations. The relationship between activity sensor values (*X_adj_* and *Y_adj_*) and the predicted proportion of time females spent feeding is represented in figure S2C, and the relationship between activity sensor values and the proportion of time males spent in sexual activities is represented in figure S2D. Similarly to the previous validations, both models had no bias (p = 0.897 for females; p = 0.229 for males) and a weak spread (p = 0.002, slope = 0.84 for females; p = 0.003, slope = 0.84 for males). The spread observed for each model means that predicted values under 0.5 are slightly underestimating actual values, whereas predicted values over 0.5 are slightly overestimating actual proportion of activity. The absence of bias means that, on average, the estimations were neither overestimated nor underestimated.

*Estimation of resting bouts*

We present the estimation of the proportion of time spent resting (step 1, Fig. S3A), the binary calculation of these data (step 2, Fig. S3B) and the result of the smoothing procedure (step 2, Fig. S3C) for a randomly chosen subsample of 24h (i.e. 1000 to 1288 recording times). Then we present the distribution of resting bout lengths according to the 2009 dataset, i.e. with a 5 min data frequency (Fig S4). We identified 134 resting bouts, among which 92.5% lasted at least 45min. The average duration of the resting bouts was 103 ±51 minutes.

**References**

1. Body G, Weladji RB, Holand Ø (2012) The recursive model as a new approach to validate and monitor activity sensors. Behav Ecol Sociobiol 66: 1531–1541.

2. Tennenhouse EM, Weladji RB, Holand Ø, Nieminen M (2012) Timing of reproductive effort differs between young and old dominant male reindeer. Ann Zool Fennici 49: 152–160.

3. Pearce J, Ferrier S (2000) Evaluating the predictive performance of habitat models developed using logistic regression. Ecol Model 133: 225–245.
